# A consistent methodology for forensic photogrammetry scanning of human remains using a single handheld DSLR camera

**DOI:** 10.1093/fsr/owad036

**Published:** 2023-09-28

**Authors:** Zsolt Ujvári, Máté Metzger, Gergely Gárdonyi

**Affiliations:** Directorate of Forensic Expertise, Hungarian Institute for Forensic Sciences, H-1087 Budapest Mosonyi str. 9, Hungary; Directorate of Forensic Expertise, Hungarian Institute for Forensic Sciences, H-1087 Budapest Mosonyi str. 9, Hungary; Department of Forensic Sciences, National University of Public Service, H-1083 Budapest, Ludovika square, Hungary

**Keywords:** postmortem documentation, 3D documentation, photogrammetry, optical surface scanning, cloud-to-mesh comparison

## Abstract

Due to increasingly capable algorithms and more available processing power, photogrammetry is becoming a simple, cheap, and accurate alternative to 3D optical surface scanning. With adequate application, it can be a swift documentation technique for reconstructing the geometry and body surface of deceased persons in autopsies or other forensic medical examinations. Sufficiently easy and swift 3D documentation techniques may allow 3D imaging technologies to become part of the daily routine of any forensic medical examiner or other medical personnel. This paper presents a consistent and systematic photographing methodology (as an alternative to automated or intuitive methods) for photogrammetry scanning of human remains. Although it requires manual photography, the methods presented in this paper offer a swift and easy way to capture an accurate 3D model of human remains under almost any conditions. Four different photographing procedures were tested on four subjects: (i) a systematic circular technique with 100 photos, (ii) a systematic circular technique with 50 photos, (iii) a technique loosely mimicking cameras mounted on a postmortem CT device with 98 photos, and (iv) a technique mimicking cameras mounted on a postmortem CT device with 49 photos. Measurement accuracy was tested with the aid of six adhesive control points placed at approximately the same locations on each subject. Five different distances defined by these control points were measured and compared to the measurements taken by hand. 3D photogrammetry meshes created using these techniques were also compared with point clouds acquired using a 3D laser scanner. We found that a carefully composed, tested, and systematic photographing procedure significantly improved the quality of the photogrammetry models. In terms of relative difference compared to the hand measurements, both Techniques 1 and 2 produced close results, with an average relative difference of 0.160% and 0.197% and a maximum relative difference of 0.481% and 0.481%, respectively, while models reconstructed from images taken using Techniques 3 and 4 seemed to be much less accurate, with an average relative difference of 0.398% and 0.391% and a maximum relative difference as high as 1.233% and 1.139%, respectively. This study highlights the importance of a scientifically tested methodology for obtaining high-quality 3D models in forensic applications.

**Key points:**

- Close-range photogrammetry is an easy, fast, and cheap way to acquire 3D models of human remains of forensic importance.
- A carefully composed, tested, and systematic photographing procedure has a key role and can greatly increase the quality of photogrammetry models.
- A photogrammetry-based 3D-digitalization technique could be developed and adopted as part of the daily routine during autopsies.
- Photogrammetry-based 3D models are much quicker to acquire, look more photorealistic, and are almost as accurate as certain laser scanner–based models.

## Graphical Abstract

**Graphical Abstract:**
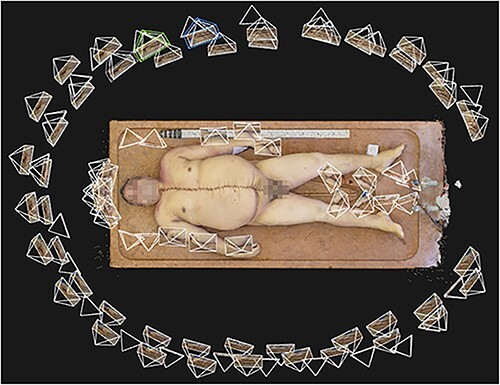

## Introduction

Currently, postmortem examination of deceased persons is documented by written reports that are usually supported by 2D photographs, as a gold standard [1–3]. Autopsy reports include, *inter alia*, comments on the deceased person’s state of preservation, demographics, circumstances of death, external examination, clothing and personal effects, medical intervention, evidence of injury, and internal examination. Forensic autopsies are slightly different from medical autopsies as the former also emphasize identification of the deceased, time of death, proper handling of evidence, recognition of injuries, and pathological conditions that may be relevant in a criminal procedure. However, even if the report is detailed, photo documentation is indispensable if further forensic examinations are carried out by different experts, and certain characteristics (e.g. toolmarks, trace material) or injuries have to be visually inspected. Size and location of these are recorded using a scale measure, while some areas are also documented as distances measured from certain anatomical points in a certain direction. Photo documentation also plays a crucial part in quality assurance: whenever a revision is necessary, often the report and photographs are the only sources of data that can be used as there is no way to reestablish the original conditions of human remains [4].

The general problem with 2D photographs is that even if a detailed autopsy report is available, we simply cannot see the “bigger picture”. Relative positions of different injuries are hard to visualize through single images; some latent injuries or characteristics might remain unnoticed, while scale bars on metric photos are usually distorted on a convex surface, which makes accurate measuring difficult. A complex 3D model, however, can be perfectly suitable to present the original condition of the body’s surface, which also makes it possible to measure any dimension or distance. The usefulness of such 3D models in forensic investigations has been clearly demonstrated [5–8]. Thus, a 3D-digitalization technique—which is easy and swift enough—should be developed and adopted as part of the daily routine (standard set of procedures followed by forensic experts during their examinations). It is important to mention that such technique would not only be useful in an autopsy setting but could also be applied on a crime scene to document the initial state, position, and surrounding environment of a body. The method proposed in this study is envisioned as a versatile tool in forensic science, with potential applications for event reconstruction and as an investigative aid. Furthermore, it could serve as a visual aid in court to support and corroborate expert testimonies, providing a clear and accurate representation of the evidence in question. Additionally, the 3D models generated through this method can be utilized by forensic scientists to re-examine the human remains at a later stage, allowing for a more comprehensive analysis and potentially uncovering new information as needed.

Until recently, accurate 3D imaging for forensic and crime scene investigation purposes has only been possible using expensive 3D laser scanners or 3D structured light scanners. Operating these devices is usually time-consuming and requires special training. On the other hand, photogrammetry—which is a technology that enables the 3D reconstruction of objects based on photographs taken from different angles—is increasingly mentioned in the literature as a possible alternative to 3D optical surface scanning [9–15]. Forensic science and crime scene investigation are two interconnected fields, which could benefit greatly from the developments of this technology.

One of the most promising aspects of forensic photogrammetry is the possibility to capture the 3D structure and surface texture of a human body. Several studies have shown that due to natural movements occurring in a living being, capturing the body of a living person requires highly specialized multi-camera rigs. These rigs are equipped with synchronized cameras, which capture images at the same time, and thus eliminate the possibility of movement between each image capture [16, 17].

However, photogrammetry reconstruction of human remains—where small movements of the subject do not hinder the reconstruction—is much easier and does not require dedicated and expensive equipment. Previous studies experimented with photogrammetry of human remains; however, they either used a mannequin and a multi-camera rig [16, 18] or the photographing process was done intuitively, without a consistent methodology [4].

It is known that the quality of a photogrammetry model is heavily influenced by the quality of the source images and the photogrammetry software [19]. Although there are some well-known general guidelines for taking pictures for photogrammetry (e.g. good illumination and high overlap between images), the importance of a consistent imaging methodology has not yet been properly evaluated.

Recently, several methods have been proposed for whole-body internal and external 3D documentation [13–27]. These techniques, however, combined internal data obtained from CT and MR with external data acquired using 3D optical surface scanning and photogrammetry. It is unlikely that these methods would widely become part of the daily routine as external data acquisition is performed during CT scanning, combined with the CT device itself, which is available only in some forensic institutes [23, 28–30]. Obviously, these techniques are also not suitable for documenting a body during forensic medical examination on a crime scene.

The primary aim of this study is to investigate the impact of different photographing procedures on the quality of 3D models acquired *via* close-range photogrammetry of human remains, with a focus on their applicability in forensic science. The specific objectives are to (i) compare the accuracy and quality of 3D models generated using various photographing and imaging techniques, (ii) evaluate the influence of the number of photographs taken on the resulting 3D model, and (iii) determine the most suitable photographing procedure for forensic applications. We tested two different photographing procedures with a variable number of images, evaluating the accuracy of each technique, while also comparing the results with 3D models acquired by a 3D laser scanner.

## Materials and methods

A Canon EOS 6D DSLR camera equipped with a Canon EF 24-70 mm f/4L IS USM lens was used to capture the images for photogrammetry reconstruction, while a Leica BLK360 Imaging Laser Scanner was used to acquire a point cloud representing the 3D structure of each test subject.

We are aware that a static laser scanner is not the most suitable equipment for scanning medium-scale objects like a human body, yet we decided to use it because it appears to be in most widespread use in Hungary on crime scenes where 3D imaging techniques are applied most frequently. Our photogrammetry software of choice was RealityCapture (Version 1.1.1.14258, Capturing Reality s.r.o, Bratislava, Slovakia). We selected this software based on our prior experience that RealityCapture worked faster and consistently yielded better results than other commercially available photogrammetry software. The point clouds acquired by the Leica scanner were exported using the Cyclone Register 360 (Version 2020.1.0, Build r17509). We used CloudCompare (www.cloudcompare.org, Version 2.11 rc1) to take measurements from the point cloud and to compare the point cloud to the photogrammetry meshes.

## Data acquisition

### Test subjects

We selected four deceased persons, three middle-aged male and one elderly female, for photogrammetry and laser scanning. The study was approved by the Institutional Research Ethics Committee of the Hungarian Institute for Forensic Sciences (case number: 29200/3915-1/2022.ált.). Written informed consent was obtained from the relatives of the deceased. Since the bodies were subject to forensic autopsies, we decided to work with post-autopsy bodies in order to eliminate any possibility of contamination or altering the condition of the bodies before the forensic autopsy was conducted. Two different photographing procedures, and two different total image count were used, yielding a total of four photogrammetry models per body. Each photoshoot was done with the bodies lying on their back only.

### Control points and measurements

Control points, represented by a 5 mm in diameter circle-shaped piece of adhesive, white paper, were placed on the top of the chin, the abdomen (adjacent to the navel), the feet (on top of the big toes), and on both hands (on the knuckle adjacent to the index finger). Five distances (chin–abdomen, abdomen–left foot, abdomen–right foot, left foot–left hand, and right foot–right hand) were measured by hand using a millimetre-scale tape measure. Measurements were taken between the centre of each control point. Each hand measurement was performed three times; average values were calculated. Tape measure was the most suitable technique for hand measurement, as those control points that designate the chosen distances were able to be connected by a straight line not crossing the body; therefore, measurement accuracy was not affected by the irregular body surfaces. Tape measurement was carried out with great care; the tape was pulled with the right strength to be straight, without any curves.

### Photography and laser scanning

Each test subject was scanned a total of five times, using four different photographing procedures and finally a Leica BLK360 Laser scanner. Two of these photographing procedures were based on our personal experimentation and consist of three photo series made from different heights, walking around the body, facing towards the centre of the body, and some additional photos of hardly visible areas. The other two photographing procedures mimic cameras mounted on computed tomography (CT) equipment with seven camera positions, facing the midsagittal plane of the body (see sections *Technique 1–Technique 4*).

Before scanning, a millimetre-scale measuring rod was placed on the autopsy table next to the test subject. Most important photography factors are listed in Table 1.

**Table 1:** Camera specifications and photographic settings used during photogrammetry scan.

Specification/settings	Parameter
Camera model	Canon EOS 6D
Sensor type	36 × 24 mm CMOS
Image resolution	5 472 × 6 348 pixels
Image format	RAW (.cr2)
Lens model	Canon EF 24-70 mm f/4L IS USM
Focal length used	24 mm
Image stabilizer	Switched off
Flash	Switched off
Tripod	Not used
ISO	1250
Lens aperture	f10
Shutter speed	1/40–1/80

A camera with a full frame sensor and a high-quality wide-angle lens is suitable for mid-scale photogrammetry. It enables users to capture a relatively wide area in great quality—even under poor lighting conditions—without significant noise and lens aberrations. The image stabilizer was turned off as it is a widely accepted general guideline that it has a negative effect on image alignment during the reconstruction of a 3D model. Lens aperture was set to f10, which enabled us to create sharp photos with a decent depth of field [31]. Aperture can and should be raised if lighting conditions are more favourable, for an even wider depth of field, provided that image quality and sharpness remain acceptable. A camera stand, flashlight, or external light source was not used in order to make the process swifter, easier, and less complicated, which makes the techniques more applicable in a real-life situation. Accordingly, to avoid camera shake by keeping shutter speed below 1/40 s, the ISO level had to be raised. Lighting conditions of the autopsy room prompted us to use a relatively high ISO value (1250), which resulted in slightly noisy images. This decision was taken because, according to our prior experience, slight image noise had no significant effect on image alignment and barely affected texture quality of the final 3D model. Photos were taken in RAW (.cr2) format and then converted to.jpg files in Adobe Lightroom CC 2015.12. The images were acquired using four different photographing techniques detailed below.

#### Technique 1

**Figure 1:**
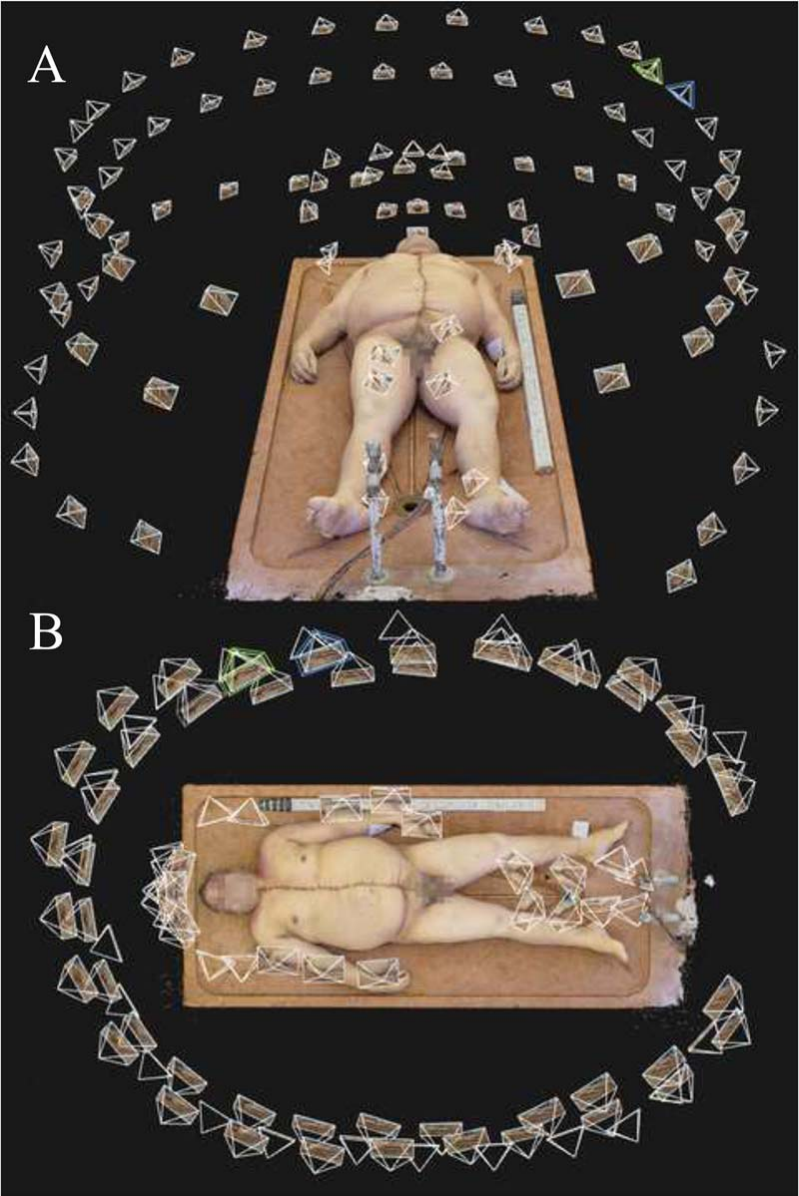
Camera positions of a photoset taken using Technique 1, as seen in the photogrammetry software: (A) front view and (B) top view.

First, we captured a total of 100 images of each body, using a technique we developed during prior experimentation through photographing well over a dozen bodies. The photos were captured according to the following methodology:

- Twenty-four images were taken walking around the body, starting from the left leg, towards the head, ending at the right leg, with the camera pointing towards the centre of the body and the axis of the camera lens closing a small angle of ~10° with the autopsy table.
- Twenty-four images were taken walking around the body, starting from the right leg, towards the head, ending at the left leg, with the camera pointing towards the centre of the body and the axis of the camera lens closing a relatively small angle of ~40° with the autopsy table.
- Twenty-four images were taken walking around the body, starting from the left leg, towards the head, ending at the right leg, with the camera pointing towards the centre of the body and the axis of the camera lens closing a larger angle of ~70° with the autopsy table.
- A total of 14 images were taken of the head, standing behind the head and facing towards the body: three pictures were taken of the top of the head, with the camera lens closing a 10°, 40°, and 70° angle with the autopsy table. Similarly, three more pictures were taken from the same angles after taking one step to the right and three more taking one step to the left from the centre of the head. Afterwards, we took one image displaying the top of the head and two images on each side displaying the neck.
- Finally, the remaining 14 pictures were taken of specific problematic areas, which generally fall outside the view of the camera, and therefore show less detail, or otherwise produce artefacts on the final model. These included four pictures of the soles, six pictures of the area between the torso and the arms (three on each side, with the camera lens closing a perpendicular angle with the autopsy table), and four pictures displaying the area between the thighs. The exact camera positions from which each image was taken can be visualized in the photogrammetry software (Figure 1).

#### Technique 2

**Figure 2:**
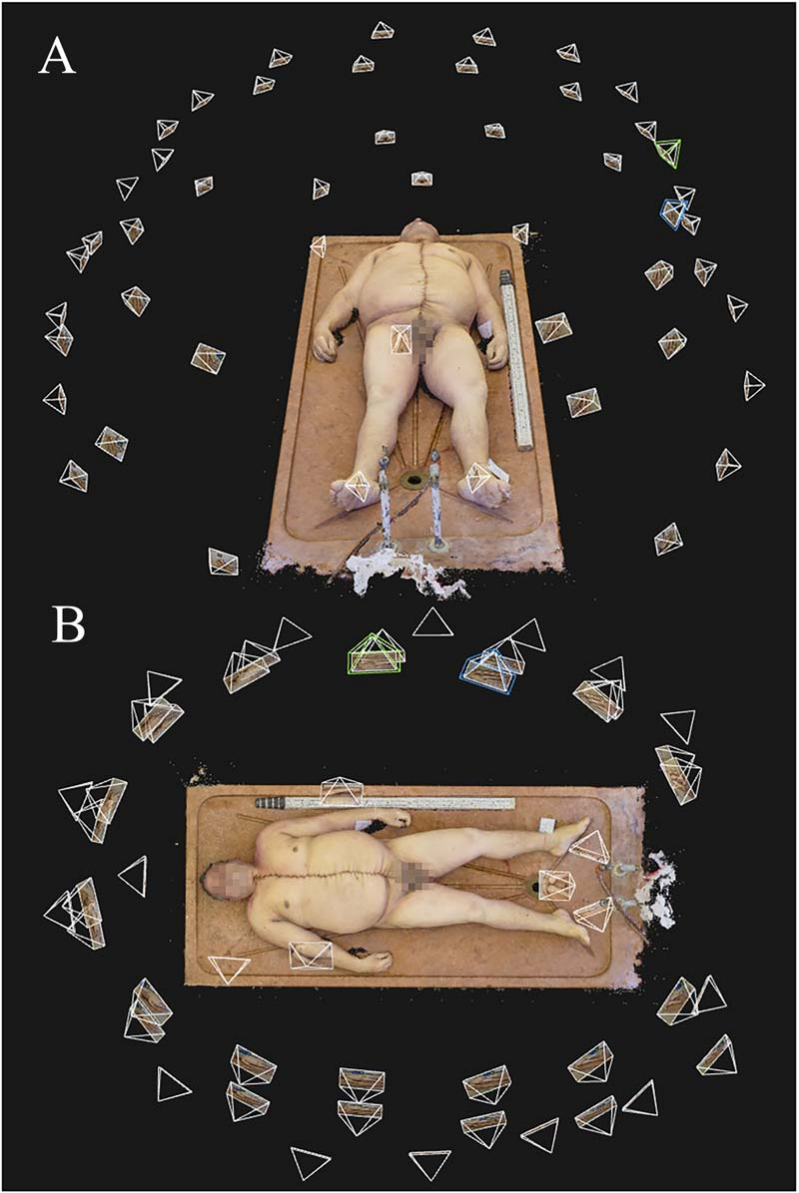
Camera positions of a photoset taken using Technique 2, as seen in the photogrammetry software: (A) front view and (B) top view.

Technique 2 followed the same procedure as Technique 1; however, we dropped the image count from 100 to 50. Accordingly, only 14 pictures were taken walking around the body on each round, only three pictures were taken of the head (one image displaying the top of the head and two displaying the neck on each side), two pictures were taken of the soles, two pictures were taken of the area between the torso and the arms (one on each side), and finally, only a single was taken of the area between the thighs (Figure 2).

#### Technique 3

This technique was loosely inspired by the possibility of combining post-mortem computed tomography equipment with a multi-camera photogrammetry rig [23], and our goal was to mimic the positions of the cameras mounted on such equipment while taking the pictures with a single handheld camera. A total of 98 photos were captured according to the following methodology:

- Fourteen pictures were taken moving from the leg of the body towards the head, moving *ca.* 15 cm between each image, with the camera lens facing the midsagittal plane of the body and the axis of the camera lens approximately parallel to the autopsy table.
- Fourteen pictures were taken moving from the head of the body towards the leg, moving *ca.* 15 cm between each image, with the camera lens facing towards the midsagittal plane of the body and the axis of the camera lens closing a small angle of ~30° with the autopsy table.
- Fourteen pictures were taken moving from the leg of the body towards the head, moving *ca.* 15 cm between each image, with the camera lens facing the midsagittal plane of the body and the axis of the camera lens closing a small angle of ~60° with the autopsy table.
- Another 42 pictures were taken, repeating the process on the opposite side of the autopsy table.
- Finally, 14 pictures were taken moving from the head towards the legs, leaning above the body, directly from above, with the axis of the camera lens approximately perpendicular to the autopsy table.

**Figure 3:**
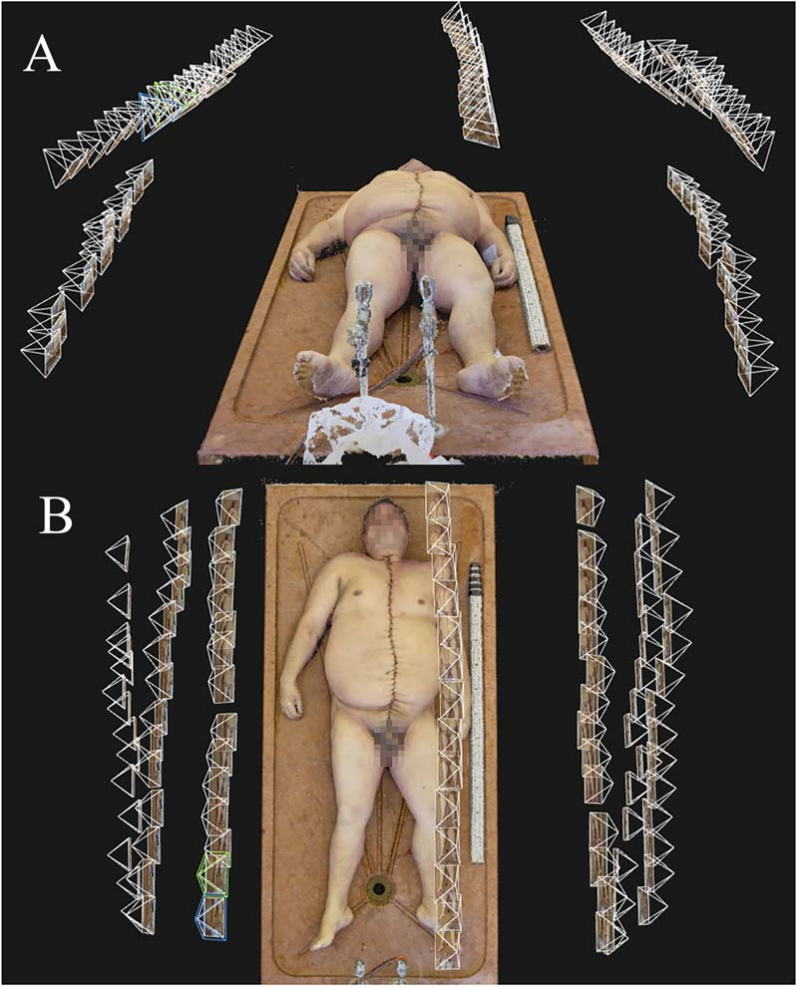
Camera positions of a photoset taken using Technique 3, as seen in the photogrammetry software: (A) front view and (B) top view.

Following the procedure, the camera positions align themselves in seven roughly straight lines, forming a semicylinder around the body as seen on Figure 3.

#### Technique 4

**Figure 4:**
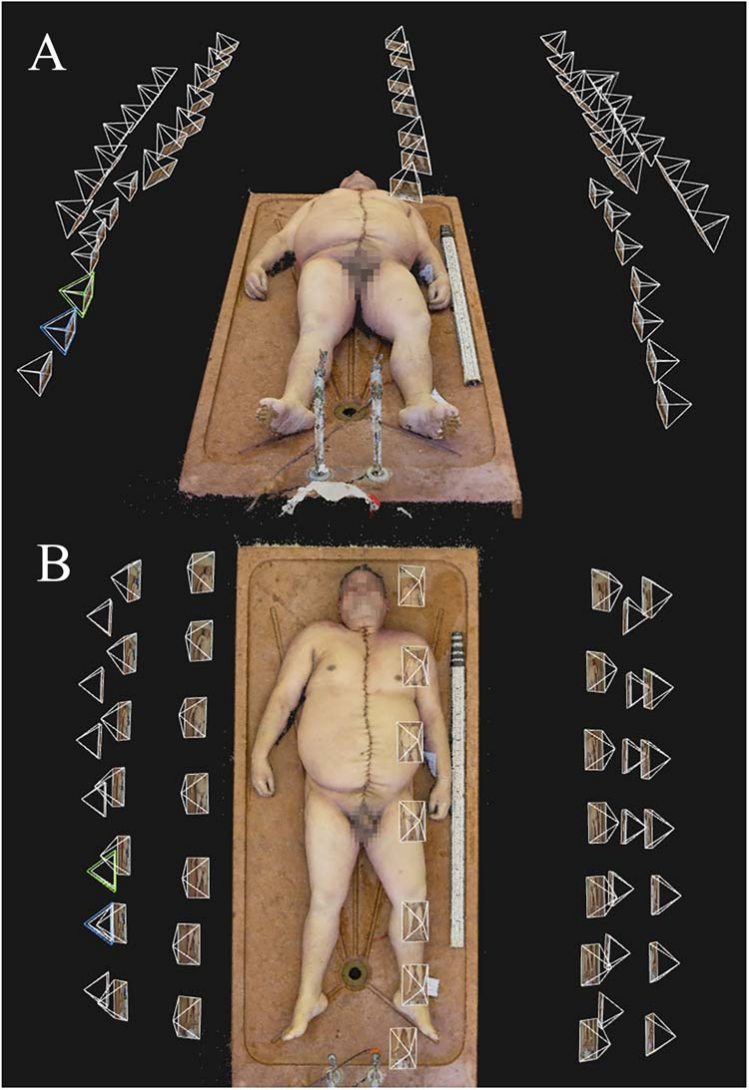
Camera positions of a photoset taken using Technique 4, as seen in the photogrammetry software: (A) front view and (B) top view.

Technique 4 followed the same procedure as Technique 3; however, we dropped the image count from 98 to 49. Accordingly, only seven pictures were taken in each line (Figure 4).

#### Laser scanning

The bodies were subsequently scanned from four different positions—one near the head, in the midsagittal plane of the body, two on each side, at the legs and torso—using the Leica BLK360 laser scanner, which was operated by a trained crime scene investigator.

#### Temporal factor

The time required to apply these techniques is a deciding factor in determining whether it is practical to apply any of these techniques in real forensic cases. Therefore, the duration of each photoshoot, as well as the time spent operating the laser scanner, was recorded and is shown in Table 2.

**Table 2:** Approximate duration of photoshoots and laser scanning of each subject in minutes.

Subject	Technique 1	Technique 2	Technique 3	Technique 4	Laser scanning
1	8	6	7	5	20
2	9	5	7	4	20
3	9	5	7	5	20
4	8	5	6	5	20

## Postprocessing

### Photogrammetry reconstruction

The .jpg image files acquired using Techniques 1–4 and converted using Adobe Lightroom were imported into RealityCapture. Images were aligned using the default alignment settings, except downscaling of images was set to 1 (no downscaling) for maximum quality. After image alignment, the reconstruction region was adjusted using RealityCapture’s box selection tool. The reconstruction area included the body and the autopsy table.

Mesh reconstruction was completed in normal detail, using the default settings (including a default image downscale factor of 2). The models were textured with an 8 k texture, without downscaling the source images. Computation time for the 100 and 98 image models was *ca.* 20 min, while it was *ca.* 9 min for the 50 and 49 image models. Final models were scaled based on two known distances, using RealityCapture’s “Define distance” tool. The first known distance was the millimetre-scale measuring rod laid next to the subjects. First, two control points were placed on the scale bar exactly 1 000 mm apart from each other. Control points were placed very carefully with considerable zoom to match the lining of the scale bar as closely as possible. Then we used the standard internal width of the autopsy table as a second scale (perpendicular to the first scale). Two well-defined points were selected on the internal edges of the table (by marking an equal distance downwards from the top on both inner rims of the table in the photogrammetry software), the distance between which was measured by hand in the autopsy room three times and resulted exactly 860 mm each time, which corresponds to the standard internal width value of the table. In the software, two control points were placed on these well-defined points of the table the same way as it was described in case of the measuring rod. The 1 000 mm distance defined on the measuring rod and the 860 mm distance defined on the autopsy table were set as reference, based on which RealityCapture scaled the models automatically. Then, distances between each relevant control points were measured in the software on each scaled model, and eventually, measurements were recorded. The final models were exported from the software as an .obj file.

### Postprocessing of laser scan data

Laser scan data were processed using the Leica Cyclone Register 360 imaging software; then, the scaled point cloud was exported as a .ptx file with RGB colour information. The point cloud was imported into CloudCompare. Distances between each relevant control points were measured using CloudCompare’s point picking tool; then, measurements were recorded.

### Comparison of photogrammetry and laser scanned models

For each test subject, the .obj files containing the photogrammetry models, as well as the corresponding laser scanned point cloud were imported into CloudCompare. The point cloud was cleaned using CloudCompare’s Statistical Outlier Removal (SOR) tool, using the default settings. Each photogrammetry model was first roughly aligned with the point cloud manually; then, CloudCompare’s registration tool was used to finely register the mesh with the point cloud, thus creating a 3D superposition of the point cloud and the mesh. Afterwards, the autopsy table was removed from the registered models using CloudCompare’s segmentation tool. Then, we re-registered the segmented point cloud with the segmented photogrammetry mesh using the mesh as a reference, for a more perfect fit. Finally, we applied CloudCompare’s cloud-to-mesh (C2M) tool with the default settings to compute the signed and the unsigned (absolute) distances between the point cloud and the photogrammetry mesh reconstructed from images acquired by Techniques 1–4.

## Results

### Model properties and control point measurements

The triangle and vertex count of each reconstructed model can be seen in Table 3. The measurements taken from each photogrammetry model in RealityCapture, as well as from the laser scanned point cloud in CloudCompare, can be seen in Table 4.

**Table 3:** Properties of the final photogrammetry meshes ×10^6^.

	Technique 1	Technique 2	Technique 3	Technique 4
Subject 1				
Triangle count	17.3	6.3	13.0	10.1
Vertex count	8.7	3.3	6.5	5.1
Subject 2				
Triangle count	17.6	6.5	12.3	9.7
Vertex count	8.9	3.2	6.2	4.9
Subject 3				
Triangle count	17.7	7.0	13.1	10.6
Vertex count	8.9	3.5	6.6	5.3
Subject 4				
Triangle count	15.9	6.8	12.5	9.8
Vertex count	8.0	3.4	6.3	4.9

**Table 4:** Distances between each control point in millimetres, as measured with a tape measure (hand measurements), in the photogrammetry software (photogrammetry meshes), as well as CloudCompare (Leica BLK360 scans).

	Tape measurements	Technique 1	Technique 2	Technique 3	Technique 4	Leica BLK 360
Subject 1						
Chin–abdomen	681	681	681	678	679	684
Abdomen–left foot	1 064	1 063	1 062	1 056	1 057	1 059
Abdomen–right foot	1 054	1 052	1 051	1 041	1 042	1 055
Left foot–left hand	905	907	906	900	901	907
Right foot—right hand	958	962	962	952	955	960
Subject 2						
Chin—abdomen	622	620	621	620	620	619
Abdomen—left foot	1 045	1 043	1 044	1 039	1 041	1 049
Abdomen—right foot	1 025	1 025	1 026	1 020	1 023	1 031
Left foot—left hand	873	873	874	871	872	875
Right foot—right hand	962	964	965	960	963	970
Subject 3						
Chin—abdomen	557	557	558	557	556	558
Abdomen—left foot	1 048	1 049	1 050	1 045	1 043	1 047
Abdomen—right foot	1 054	1 055	1 056	1 050	1 048	1 062
Left foot—left hand	860	860	861	858	856	856
Right foot—right hand	933	932	932	927	927	934
Subject 4						
Chin—abdomen	619	620	620	619	618	620
Abdomen—left foot	817	818	818	815	814	818
Abdomen—right foot	831	834	835	829	827	836
Left foot—left hand	821	824	824	822	821	823
Right foot—right hand	787	789	789	784	783	787

We compared each software measurement with the measurements taken by hand in the autopsy room. We calculated the relative change (distance relative to hand measurements) for each measurement, using the formula


$$ C=\frac{x_2-{x}_1}{x_1}\times 100 $$

where *x*_2_ represents the hand measurement and *x*_1_ represents the software measurement. Based on the results, the mean squared error (MSE) was also calculated. The results are summarized in Table 5.

**Table 5:** Relative difference (percentage) and mean squared error (MSE) of the software measurements taken on the models reconstructed using each technique.

Subject	Chin–abdomen	Abdomen–left foot	Abdomen–right foot	Left foot–left hand	Right foot–right hand	MSE	Mean relative change (%)
Technique 1 error							
1	0.000	0.094	0.190	−0.221	−0.418	5.000	0.184
2	0.322	0.191	0.000	0.000	−0.208	2.400	0.144
3	0.000	−0.095	−0.095	0.000	0.107	0.600	0.059
4	−0.162	−0.122	−0.361	−0.365	−0.254	4.800	0.253
Mean						3.200	0.160
Technique 2 error							
1	0.000	0.188	0.285	−0.110	−0.418	6.000	0.200
2	0.161	0.096	−0.098	−0.115	−0.312	2.600	0.156
3	−0.180	−0.191	−0.190	−0.116	0.107	2.200	0.157
4	−0.162	−0.122	−0.481	−0.365	−0.254	6.200	0.277
Mean						4.250	0.197
Technique 3 error							
1	0.441	0.752	1.233	0.552	0.626	60.600	0.721
2	0.322	0.574	0.488	0.229	0.208	14.600	0.364
3	0.000	0.286	0.380	0.233	0.643	13.000	0.308
4	0.000	0.245	0.241	−0.122	0.381	3.600	0.198
Mean						22.950	0.398
Technique 4 error							
1	0.294	0.658	1.139	0.442	0.313	44.400	0.569
2	0.322	0.383	0.195	0.115	−0.104	5.200	0.224
3	0.180	0.477	0.569	0.465	0.643	22.800	0.467
4	0.162	0.367	0.481	0.000	0.508	8.400	0.304
Mean						20.200	0.391
Leica BLK360 Laser Scanner error							
1	−0.441	0.470	−0.095	−0.221	−0.209	8.600	0.287
2	0.482	−0.383	−0.585	−0.229	−0.832	25.800	0.502
3	−0.180	0.095	−0.759	0.465	−0.107	16.600	0.321
4	−0.162	−0.122	−0.602	−0.244	0.000	6.200	0.226
Mean						14.300	0.334

In terms of relative difference compared to the hand measurements, both Techniques 1 and 2 produced close results, with an average relative difference of 0.160% and 0.197% and a maximum relative difference of 0.418% and 0.481%, respectively. On the other hand, models reconstructed from images taken using Techniques 3 and 4 seem to be much less reliable, with an average relative difference of 0.398% and 0.391% and a maximum relative difference as high as 1.233% and 1.139%, respectively.

The measurements taken from the point cloud reconstructed with the Leica BLK360 laser scanner show a mean relative distance of 0.334% and a maximum relative distance of 0.832%, which is mostly within the advertised short-distance accuracy of 6 mm on all measurements.

### Model quality relative to the laser scanned point cloud

The results of the cloud-to-mesh comparison are displayed in Table 6. The mean signed distance between the laser scanned point cloud and the photogrammetry meshes is between ~−1.29 and −1.53 mm, whereas the mean absolute distances ranged from 3.90 to 4.07 mm, which is consistent with the results of some previous studies [4, 23].

**Table 6:** Results of the signed and absolute cloud-to-mesh comparison (using the mesh as reference) for each subject and technique, shown in millimetres.

Subject	Technique 1		Technique 2		Technique 3		Technique 4	
	Signed	Absolute	Signed	Absolute	Signed	Absolute	Signed	Absolute
1	−1.003	3.293	−1.077	3.287	−0.785	3.175	−0.824	3.184
2	−1.800	4.295	−1.753	4.420	−1.713	4.265	−1.409	4.236
3	−1.677	4.116	−1.719	4.191	−1.626	4.116	−1.547	4.035
4	−1.508	4.291	−1.550	4.370	−1.468	4.154	−1.385	4.143
Mean	−1.497	3.999	−1.525	4.067	−1.398	3.928	−1.291	3.900

**Figure 5:**
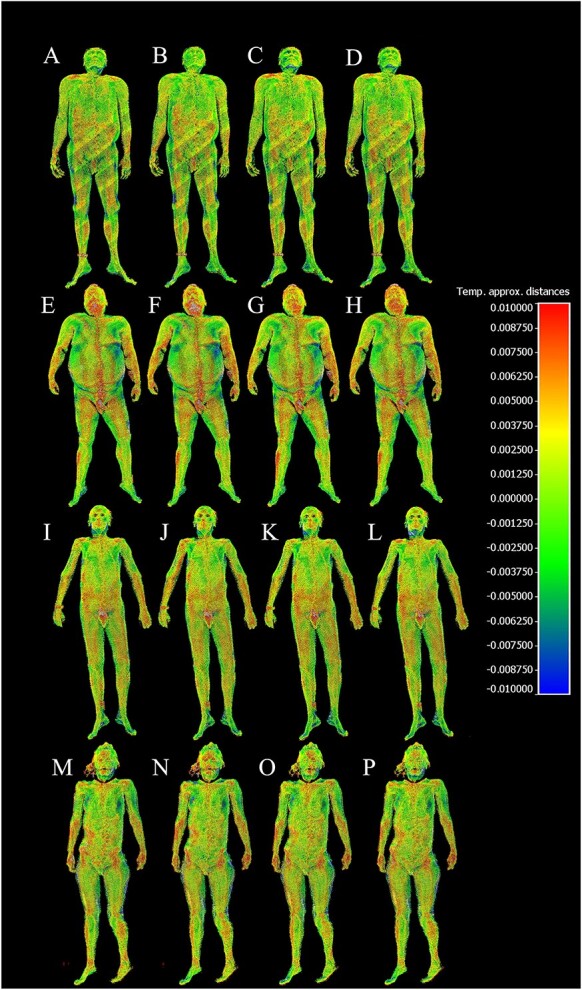
Cloud-to-mesh signed distances between the laser scanned point cloud acquired by a BLK360 scanner and photogrammetry models acquired on different subjects (S1–4), using different techniques (T1–4); values of the scale on the right are given in metres: (A) S1-T1, (B) S1-T2, (C) S1-T3, (D) S1-T4, (E) S2-T1, (F) S2-T2, (G) S2-T3, (H) S2-T4, (I) S3-T1, (J) S3-T2, (K) S3-T3, (L) S3-T4, (M) S4-T1, (N) S4-T2, (O) S4-T3, and (P) S4-T4.

**Figure 6:**
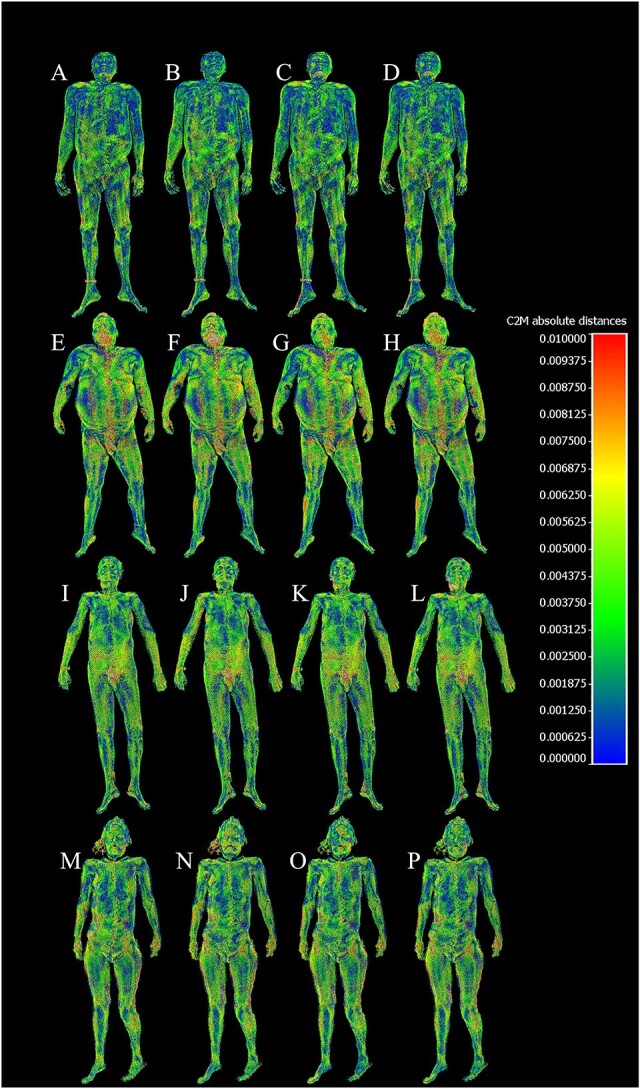
Cloud-to-mesh absolute distances between the laser scanned point cloud acquired by a BLK360 scanner and photogrammetry models acquired on different subjects (S1–4), using different techniques (T1–4); values of the scale on the right are given in metres: (A) S1-T1, (B) S1-T2, (C) S1-T3, (D) S1-T4, (E) S2-T1, (F) S2-T2, (G) S2-T3, (H) S2-T4, (I) S3-T1, (J) S3-T2, (K) S3-T3, (L) S3-T4, (M) S4-T1, (N) S4-T2, (O) S4-T3, and (P) S4-T4.

The differences between the laser scanned point cloud and the photogrammetry meshes can be visualized as a coloured heatmap in CloudCompare to gain more insight about which areas of the bodies contribute more to the differences, as seen on Figures 5 and 6.

Figures 5 and 6 provide a visual representation of the cloud-to-mesh signed and absolute distances, respectively, between the laser-scanned point cloud acquired by a BLK360 scanner and photogrammetry models acquired on different subjects (S1–4) using different techniques (T1–4). In Figure 5, zero error is represented by light green, with positive distances turning towards red and negative distances turning towards dark blue. In Figure 6, zero error is depicted as dark blue, and as the absolute error increases, it transitions to green and then red. These figures help to illustrate the differences in model quality between the various techniques and emphasize the importance of selecting the appropriate technique for the desired level of accuracy and detail in forensic applications.

Some areas of difficulty with higher error rates were observed in regions covered with hair or body hair, as well as in areas with more complex geometry, such as the eye sockets and the groin.

## Discussion

Before delving into the discussion on the differences between the techniques, it is crucial to note a few key observations. Techniques 1 and 2 appeared to produce more accurate results on top of the head, between the arm and the torso, between the thighs, and the soles. We believe that the superior performance of Techniques 1 and 2 could be attributed to their careful composition, which aimed to cover as much body area as possible from a wider range of angles and account for harder-to-reach areas. Techniques 3 and 4 were slightly faster than Techniques 1 and 2, and no significant differences were observed between Techniques 1 and 2 or between Techniques 3 and 4, which were of the same type. All techniques struggled in some areas, such as those with hair and more complex geometry. Although human error is a factor in photogrammetry, we strived to minimize its impact by employing an operator with significant experience in photography and photogrammetry. Furthermore, we believe that the effect of any potential human error is reduced by the relatively large number of images and the involvement of multiple subjects.

### Measurement accuracy

As expected, the results seem to indicate that photography procedure has a very significant impact on the quality of the final photogrammetry models. Working with Techniques 1 and 2 resulted in a 3D model with dimensions much closer to the measurements taken by hand in the autopsy room. It is possible, that Techniques 1 and 2 produced better results because the camera positions were chosen carefully and individually to provide maximum coverage of the body, while Techniques 3 and 4 utilized a more systematic approach. Another possible explanation is that the reconstruction is more accurate if the camera view rotates around both the *Z* (forward) and *Y* (up) axis in 3D space as the images are taken, as it is the case with Techniques 1 and 2, and less accurate if the camera view rotates mostly only along the *Z* axis, as it is mostly the case with Techniques 3 and 4.

The measurements taken from the laser scanned point cloud exhibited a higher error than Techniques 1 and 2 when compared to the hand measurements, but this error is still within the 6 mm accuracy advertised by the manufacturer and should be interpreted with regard to the capabilities and limitations of the BLK360 scanner. Consequently, Techniques 1 and 2 produced the best error rate when compared to Techniques 3 and 4 and the BLK360 scanner.

### Number of source images

Surprisingly, the number of source images used to reconstruct the models did not have a drastic effect on the quality of the final models. The 50-image models (Technique 2) seemed to be on par with the 100-image models (Technique 1) in terms of accuracy, as well as the 49-image models (Technique 4) with the 98-image models (Technique 3). In fact, on average, the 49-image models (Technique 4) even produced slightly more accurate measurements than the 98-image models (Technique 3). However, it is worth noting that while working with only 50-image or less to reconstruct a full human body appears to be quick and practical, the overlap between subsequent images is relatively low. This may make image alignment harder for the photogrammetry software, and even a single blurry or faulty photo could potentially break the model up into multiple components, which might require additional work and advanced use of the photogrammetry software to merge. Therefore, we only recommend Technique 2 if the camera operator is experienced enough to ensure image quality and overlap, as a lower total image count can potentially amplify the effect of human error on the quality of the model.

### Model quality relative to the laser scanned point cloud

Regarding the absolute distances from the laser scanned point cloud, there seems to be no major difference between the photogrammetry models acquired by Techniques 1–4, with the average absolute distances ranging from 3.90 to 4.07 mm. We chose the laser scanned point cloud as reference because it is undisputed that laser scanning technology has better accuracy than photogrammetry. However, it is important to mention that, contrary to our expectations, the point cloud acquired using the BLK360 laser scanner also showed significant differences compared to our hand measurements, as shown in Table 5. Also, static laser scanning technology is limited by the number of scanning positions. In this study, we choose to scan each body from five different positions. However, an object with such complex geometry as a human body is difficult to capture using a limited number of positions. On the other hand, photogrammetry techniques can be adapted to the geometry of the scanned object more easily to cover areas that normally fall outside the view of the camera. This is an advantage of photogrammetry that we strived to leverage when we constructed Techniques 1 and 2.

In addition, the heatmaps generated by CloudCompare give us an indication about which parts of the body’s surface contributed more to the difference between the point cloud and the photogrammetry meshes. Generally, we experienced that areas covered by thick body hair produced artefacts on the photogrammetry meshes, which consequentially increased the distance from the point cloud in these areas.

Another important aspect of model quality is how lifelike and natural the final models look to the human eye. Naturally, this is a property which is impossible to quantify due to its subjective nature. However, we found that the final, textured photogrammetry models looked almost photorealistic, as shown in Figures 1–4. All models, even areas of the body that produced artefacts due to body hair looked surprisingly smooth and natural on the textured model, provided that no directional lighting was applied.

While all techniques produced some visual artefacts on the top of the head and between the arms and the torso, these were much less visible on models produced using Techniques 1 and 2. On all models, some artefacts appeared on the feet and the soles; however, it seems likely that these appeared due to the non-removable water pipes mounted on the edge of the autopsy table, which inevitably obstructed the view of the camera on some pictures.

### Temporal factor

Time required to capture 3D models is one of the most important factors to consider, even in an autopsy room, but even more so at a crime scene. While laser scanning from five scanning positions took ~20 min, photogrammetry techniques were much faster, as even the slowest technique (Technique 1) required 8–9 min only. It is important to mention that during photography, we took extra care of camera orientation, distances between camera positions, image overlap, and exact image count, which made the process much slower than it would be in a real-life situation. Any of the photogrammetry techniques discussed in this paper can be performed much more quickly and we suppose that, with some practice, capturing the required images of human remains with a DSLR camera would only take 2–3 min. While computation time was 9 to 20 min depending on image count, this is a highly automated process and requires minimal user intervention. We believe that time is one of the key factors that, if sufficiently shortened, would make photogrammetry techniques discussed in this paper feasible for documentation, as part of the daily routine.

### Applicability of these methods at crime scenes and in expert work

In 2011, the European Council created the vision of the “European Forensic Science 2020” concept, which includes the creation of a European Forensic Science Area (EFSA) and the development of the infrastructure of forensic sciences in Europe [32]. *Inter alia*, the goal of this idea is to adopt equivalent minimum rules for routine forensic procedures related to the collection, processing, use, and provision of data, enabling a closer cooperation between law enforcement authorities of the Member States. EFSA’s concept should have been implemented by 2020, but only a small part of the ideas has become a reality and there are no signs yet that steps will be taken to revise or extend the deadline.

Against this backdrop, it is worth to examine whether photogrammetry is suitable for application in the procedures carried out under the partially established EN ISO 21043 standard series. This issue cannot be exhaustively studied, since, as we write this paper, only standards 21043-1:2018 [33] and 21043-2:2018 [34] have been established. Nonetheless, these standards offer an opportunity for interpretation in terms of the basic definitions and the recovery and documentation of evidence.

The definition of “document” in standard 21043-1:2018 includes photographs. In terms of the recovery of evidence, standard 21043-2:2018 also defines photographs and other electronic images and footage as part of the documentation.

This technology corresponds with the concept behind the standard, reinforcing the requirements set forth in it at a strategic point; the documentation must be detailed enough so that it includes the exact location and description of the crime scene investigation, the relevant environmental conditions, the recovered items with evidential value, and the findings and location of relevant observations.

A separate, documented procedure must be established in terms of the application of photogrammetry at crime scenes, prior to the introduction of the technology. *Inter alia*, that will have to include the documentation of when, where, by whom, and under what conditions the photos were taken, as well as the number of photographs taken on the scene. Moreover, weather conditions, devices used (e.g. type and settings of the camera). and the applied photography technique (e.g. approximate angles of the photos and height where the camera was held when taking them) will also have to be documented. When establishing a standard procedure, it also should be kept in mind that photographing objects, persons, animals, plants, etc., in motion can be rather cumbersome with an uncertain result. This technology should obviously be applied predominantly in the static phase of the crime scene investigation (at the phase where the overall picture is captured); however, it might also have significance in the dynamic phase (at the phase where the traces are collected, e.g. to support recording after the recovery of a footprint suspected to be bloodstained).

Photogrammetry may also be a useful technology in expert work. Firstly, additional information can be gained when delivering the expert report based on images created with photogrammetry after taking photographs at the crime scene (e.g. a 3D footprint). Secondly, the expert can also apply photogrammetry to present the examined traces or the derived conclusions in a more illuminating manner to the court in cases where the examinations performed by the expert damage the item recovered at the crime scene (e.g. the examination of locks or bones). The expert might also request the investigative authority to provide access to the photogrammetry photosets created of a recovered item to create a 3D model that can support the forensic testimony (which might even be possible without handing over the item itself).

The issue of taking photos of evidence also arise in relation to the handling of items recovered at the crime scene. Photographs can be taken by the members of the investigative authority when confiscating, creating, or receiving an item, which can also be useful if the item is handed over to the owner or to another authority. Photogrammetry has the advantage over conventional photographs to provide the authorities, the experts, or the defence attorneys with the opportunity of a full, 3D examination without looking at the item or even if the item itself was destroyed, also providing data on dimensions where necessary.

## Summary

Results are briefly summarized in Table 7. Those properties and values that we considered best are highlighted in bold.

**Table 7:** Different properties and calculated values of photogrammetry techniques and 3D laser scanning based on four subjects.

Parameters	Technique 1	Technique 3	Technique 4	Laser scanner
Photos taken	100	98	**49**	N/A
Duration (min)	8–9	6–7	**4–5**	20
Accuracy (mean relative change in percentage)	**0.160**	0.398	0.391	0.334
Accuracy (mean squared error)	**3.200**	22.950	20.200	14.300
Model quality (cloud-to-mesh signed distance, in mm)	−1.497	−1.398	−1.291	N/A
Model quality (cloud-to-mesh absolute distance, in mm)	3.999	3.928	3.900	N/A
Visible model quality (presence of 3D artefacts and untextured areas)	**Good** (less artefacts on parts outside the line of sight, mostly on arms and thighs)	Average (artefacts on parts outside the line of sight, soles and head)	Average (artefacts on parts outside the line of sight, soles and head)	Average (artefacts on parts outside the line of sight, soles and head)
Texture quality	**Good**	**Good**	**Good**	Average

Best properties and values are highlighted in bold. N/A: not applicable.

Regarding data acquisition time, static, long-range laser scanning is still a slow operation compared to close and mid-range photogrammetry. Obviously, capturing less images takes less time, and it appears that image count does not have a serious effect on measurement accuracy or model quality relative to the point cloud acquired by the BLK360 scanner. Capturing ~50 photos of human remains seems to be adequate; however, with less photos and relatively low overlap, there is a chance that a few bad quality photos could prevent 3D reconstruction. Our results clearly support that the photography procedure has a serious effect on measurement accuracy and model quality. Based on relative change compared to hand measurements and the MSE, circular methods (Techniques 1–2) resulted in much more accurate models with less artefacts than the CT-inspired methods (Techniques 3–4). Obviously, there is a trade-off between model accuracy, temporal factor, manpower, and cost. The original CT photogrammetry scanning method—which we intended to mimic—requires a CT and a set of cameras. It is totally automated and hence does not require human intervention while taking photos; therefore, it lacks human errors like camera shake or blurry images and therefore can ensure better image quality. However, it is not flexible and customizable enough to ensure that hardly visible areas of the body are also covered, which can result in lower model quality. On the contrary, the circular photogrammetry techniques (Techniques 1–2) are cost-effective, require only a single handheld camera, and result in better model quality, but may suffer from human errors and require more time than the CT-inspired method.

Moreover, in our case, accuracy of circular methods has proven to be better than that of the BLK360 laser scanner. However, this result is in no way any indication that the techniques proposed in this paper are more accurate than 3D optical surface scanning technology in general, as accuracy can greatly depend on the type of the 3D optical surface scanner used for data acquisition, as well as the number of scanning positions and the software used to postprocess the scan. A mid-scale handheld 3D optical surface scanner could produce more accurate models of smaller objects than a 360° static scanner, and more research is needed to compare models acquired by a handheld 3D optical surface scanner to photogrammetry models.

Comparing photogrammetry 3D meshes to point clouds obtained by the laser scanner, we experienced some variation in signed and absolute distances. For a better interpretation of the correlation between model quality and photogrammetry methods and image count, a bigger dataset and a finer 3D optical surface scanning method would be necessary [9].

On balance, circular photogrammetry methods have proven to be the best of all methods tested, CT-inspired methods have several disadvantages, especially artefacts and missing textures; consequentially, model quality seems to be lower. Application of these fast and accurate techniques at both autopsies and crime scenes would allow swift and high-quality documentation—with much more information compared to standard photographs—therefore, it could be a cornerstone of quality assurance in the future.

## Conclusions

As a result of increasingly capable algorithms and more available processing power, photogrammetry has gained popularity as a technique with significant potential for scientific applications. Close-range photogrammetry is faster and cheaper than using expensive 3D optical surface scanners, and the 3D models acquired *via* photogrammetry could potentially be used for forensic purposes. However, the quality of photogrammetry models can vary greatly based on the quality of source images, equipment, photogrammetry software used, and the methodology applied during image acquisition. In this study, we have shown the importance of the latter and established that a carefully composed, tested, and systematic photographing procedure can greatly increase the quality of photogrammetry models.

We believe that photogrammetry could be an immensely useful technique in various fields of forensic science, such as injury and wound analysis, and even crime scene documentation. For example, accurate 3D models of human remains could provide valuable information for determining the cause and manner of death, preserve details of the body that may not seem significant at the time of the autopsy, or even support victim identification. Moreover, comprehensive 3D models of human remains could assist in analysing and presenting complex spatial relationships between injuries or other relevant pieces of evidence. However, scientifically tested methodology is required to maximize the quality of 3D models and discover the limits of how accurate a photogrammetry model can be. This is especially important if the photogrammetry models are to be admitted at court as supplementary evidence, where reliability and precision are crucial.

## References

[1] DiMaio VJM , Molina DK. DiMaio’s forensic pathology: practical aspects of criminal and forensic investigations, 2nd edn. Boca Raton (FL): CRC Press, 2001.

[2] Prahlow JA . Forensic pathology for police, death investigators, attorneys, and forensic scientists. New York (NY): Springer, 2010

[3] Shkrum MJ , Ramsay DA. Forensic pathology of trauma: forensic science and medicine. Totova (NJ): Humana Press, 2006.

[4] Urbanová P , Hejna P, Jurda M. Testing photogrammetry-based techniques for three-dimensional surface documentation in forensic pathology. Forensic Sci Int. 2015;250:77–86. 25818581 10.1016/j.forsciint.2015.03.005

[5] Thali MJ , Braun M, Bruschweiler W, et al. Matching tire tracks on the head using forensic photogrammetry. Forensic Sci Int. 2000;113:281–287. 10978638 10.1016/s0379-0738(00)00234-6

[6] Buck U , Naether S, Braun M, et al. Application of 3D documentation and geometric reconstruction methods in traffic accident analysis: with high resolution surface scanning, radiological MSCT/MRI scanning and real data based animation. Forensic Sci Int. 2007;170:20–28. 16997523 10.1016/j.forsciint.2006.08.024

[7] Buck U , Naether S, Rass B, et al. Accident or homicide—virtual crime scene reconstruction using 3D methods. Forensic Sci Int. 2013;225:75–84. 22727689 10.1016/j.forsciint.2012.05.015

[8] Villa C , Flies MJ, Jacobsen C. Forensic 3D documentation of bodies: simple and fast procedure for combining CT scanning with external photogrammetry data. J Forensic Radiol Imaging. 2018;12:e2–e7.

[9] Buck U , Buße K, Campana L, et al. Validation and evaluation of measuring methods for the 3D documentation of external injuries in the field of forensic medicine. Int J Leg Med. 2018;132:551–561. 10.1007/s00414-017-1756-6 29260394

[10] Rahaman H , Champion E, Bekele M. From photo to 3D to mixed reality: a complete workflow for cultural heritage visualisation and experience. Digit Appl Archaeol Cult Heritage. 2019;13:e00102–e00112.

[11] González-Merino R , Fraile AD, Pérez JA, et al. Validation of photogrammetry techniques performed on two lead ingots assigned to Linares Historical Heritage. Procedia Manuf. 2017;13:1405–1412.

[12] Siebke I , Campana L, Ramstein M, et al. The application of different 3D-scan-systems and photogrammetry at an excavation—a Neolithic dolmen from Switzerland. Digit Appl Archaeol Cult Heritage. 2018;10:e00078–e00011.

[13] Leipner A , Baumeister R, Thali MJ, et al. Multi-camera system for 3D forensic documentation. Forensic Sci Int. 2016;261:123–128. 26921815 10.1016/j.forsciint.2016.02.003

[14] Grabherr S , Egger C, Vilarino R, et al. Modern post-mortem imaging: an update on recent developments. Forensic Sci Res. 2017;2:52–64. 30483621 10.1080/20961790.2017.1330738 PMC6197109

[15] Cuerrier-Richer E . Missing and murdered indigenous women and girls in Canada: a new population affinity assessment technique to aid in identification using 3D technology. Forensic Sci Res. 2021;7:427–439. 10.1080/20961790.2021.2023417 PMC9639536 36353333

[16] Michienzi R , Meier S, Ebert LC, et al. Comparison of forensic photo-documentation to a photogrammetric solution using the multi-camera system “Botscan”. Forensic Sci Int. 2018;288:46–52. 29715622 10.1016/j.forsciint.2018.04.012

[17] Leipner A , Obertová Z, Wermuth M, et al. 3D mug shot—3D head models from photogrammetry for forensic identification. Forensic Sci Int. 2019;300:6–12. 31059949 10.1016/j.forsciint.2019.04.015

[18] Koller S , Ebert LC, Martinez RM, et al. Using virtual reality for forensic examinations of injuries. Forensic Sci Int. 2019;295:30–35. 30554020 10.1016/j.forsciint.2018.11.006

[19] Kingsland K . Comparative analysis of digital photogrammetry software for cultural heritage. Digit Appl Archaeol Cult Heritage. 2020;18:e00157–e00110.

[20] Kottner S , Schaerli S, Fürst M, et al. VirtoScan-on-rails—an automated 3D imaging system for fast post-mortem whole-body surface documentation at autopsy tables. Forensic Sci Med Pathol. 2019;15:198–212. 30850988 10.1007/s12024-019-00095-5

[21] Kottner S , Schulz MM, Berger F, et al. Beyond the visible spectrum—applying 3D multispectral full-body imaging to the VirtoScan system. Forensic Sci Med Pathol. 2021;17:565–576. 34533694 10.1007/s12024-021-00420-x PMC8629877

[22] Kottner S , Ebert L, Ampazoni G, et al. A mobile, multi camera setup for 3D full body imaging in combination with post-mortem computed tomography procedures. In: D’Apuzzo N, editor. Proceedings of 7th International Conference on 3D Body Scanning Technologies. Lugano, Switzerland, 2016 Nov 30 – Dec 1, Lugano (Switzerland): Hometrica Consulting. p. 53–60.

[23] Kottner S , Ebert LC, Ampanozi G, et al. VirtoScan—a mobile, low-cost photogrammetry setup for fast post-mortem 3D full-body documentations in X-ray computed tomography and autopsy suites. Forensic Sci Med Pathol. 2017;13:34–43. 28144846 10.1007/s12024-016-9837-2

[24] Thali MJ , Braun M, Buck U, et al. VIRTOPSY—scientific documentation, reconstruction and animation in forensic: individual and real 3D data based geo-metric approach including optical body/object surface and radiological CT/MRI scanning. J Forensic Sci. 2005;50:428–442. 15813556

[25] Thali MJ , Braun M, Wirth J, et al. 3D surface and body documentation in forensic medicine: 3-D/CAD photogrammetry merged with 3D radiological scanning. J Forensic Sci. 2003;48:1356–1365. 14640285

[26] Buck U , Naether S, Thali M. External body documentation. In: Thali M, Dirnhofer R, Vock P, editors. The virtopsy approach: 3D optical and radiological scanning and reconstruction in forensic medicine. Boca Raton (FL): CRC Press, 2009. p. 51–60.

[27] Buck U , Naether S, Thali MJ. Virtopsy as a multi-tool approach. In: Thali M, Dirnhofer R, Vock P, editors. The virtopsy approach: 3D optical and radiological scanning and reconstruction in forensic medicine. Boca Raton (FL): CRC Press, 2009. p. 389–442.

[28] Ebert LC , Ptacek W, Naether S, et al. Virtobot—a multi-functional robotic system for 3D surface scanning and automatic post mortem biopsy. Int J Med Robotics Comput Assist Surg. 2010;6:18–27. 10.1002/rcs.285 19806611

[29] Ebert LC , Flach P, Schweitzer W, et al. Forensic 3D surface documentation at the Institute of Forensic Medicine in Zurich—workflow and communication pipeline. J Forensic Radiol Imaging. 2016;5:1–7.

[30] Ebert LC , Ptacek W, Breitbeck R, et al. Virtobot 2.0: the future of automated surface documentation and CT-guided needle placement in forensic medicine. Forensic Sci Med Pathol. 2014;10:179–186. 24474435 10.1007/s12024-013-9520-9

[31] Nocerino E , Menna F, Verhoeven GJ. Good vibrations? How image stabilisation influences photogrammetry. Int Arch Photogramm Remote Sens Spatial Inf Sci. 2022;46:398.

[32] Nogel M , Czebe A, Kovács G, et al. A work in progress—accreditation of forensic DNA laboratories as a part of the “European forensic science area 2020 (EFSA 2020)” concept. Forensic Sci Int Genet Suppl Ser. 2019;7:836–837.

[33] ISO 21043-1:2018 Forensic sciences—Part 1: Terms and definitions.

[34] ISO 21043-2:2018 Forensic sciences—Part 2: Recognition, recording, collecting, transport and storage of items.

